# Meet OLAF, a Good Friend of the IAPS! The Open Library of Affective Foods: A Tool to Investigate the Emotional Impact of Food in Adolescents

**DOI:** 10.1371/journal.pone.0114515

**Published:** 2014-12-09

**Authors:** Laura Miccoli, Rafael Delgado, Sonia Rodríguez-Ruiz, Pedro Guerra, Eduardo García-Mármol, M. Carmen Fernández-Santaella

**Affiliations:** 1 Department of Personality, University of Granada, Granada, Spain; 2 Department of Physical Education and Sports, University of Granada, Granada, Spain; The University of Queensland, Australia

## Abstract

In the last decades, food pictures have been repeatedly employed to investigate the emotional impact of food on healthy participants as well as individuals who suffer from eating disorders and obesity. However, despite their widespread use, food pictures are typically selected according to each researcher's personal criteria, which make it difficult to reliably select food images and to compare results across different studies and laboratories. Therefore, to study affective reactions to food, it becomes pivotal to identify the emotional impact of specific food images based on wider samples of individuals. In the present paper we introduce the Open Library of Affective Foods (OLAF), which is a set of original food pictures created to reliably select food pictures based on the emotions they prompt, as indicated by affective ratings of valence, arousal, and dominance and by an additional food craving scale. OLAF images were designed to allow simultaneous use with affective images from the International Affective Picture System (IAPS), which is a well-known instrument to investigate emotional reactions in the laboratory. The ultimate goal of the OLAF is to contribute to understanding how food is emotionally processed in healthy individuals and in patients who suffer from eating and weight-related disorders. The present normative data, which was based on a large sample of an adolescent population, indicate that when viewing affective non-food IAPS images, valence, arousal, and dominance ratings were in line with expected patterns based on previous emotion research. Moreover, when viewing food pictures, affective and food craving ratings were consistent with research on food cue processing. As a whole, the data supported the methodological and theoretical reliability of the OLAF ratings, therefore providing researchers with a standardized tool to reliably investigate the emotional and motivational significance of food. The OLAF database is publicly available at zenodo.org.

## Introduction

In the current globalized society, as a result of the dramatic coexistence of disorders related to weight and eating behaviors [1], there is widespread scientific interest in understanding how healthy and pathological populations react to food cues. Recently, researchers have begun to focus on the emotional significance of food in obesity, as well as in eating disorders, and few studies have used food and affective images to investigate the emotional impact of food in the laboratory [2]–[4]. However, a major difficulty is that the primary method for selecting food images is based on a researcher's personal criteria. Therefore, even if it is well known that some food categories, namely foods high in both fat and sugar, are more appealing and likeable compared with other foods [5], it is difficult for researchers to know a priori which specific food pictures will be motivationally and emotionally more (or less) engaging compared with other food pictures in large groups of individuals. In this context, it is noteworthy that during a recent conference of the Nutrition Society, researchers have openly acknowledged the need to develop standardized sets of food images [6]. In the present research, we created a database of original food pictures and assessed how a large group of participants rated how they felt looking at each image. Our main goal was to provide researchers with a standardized set of affectively rated food images that could simultaneously be used with affective pictures from the International Affective Pictures System/IAPS [7].

### The food cue reactivity paradigm

In research on eating pathology, the “food cue reactivity paradigm” [8] has been commonly employed to investigate subjective, behavioral, and physiological reactions to food images in healthy controls and individuals affected by eating disorders and obesity (e.g., [9]–[16]). Typically, participants' reactions to images of high-calorie food are compared with the reactions while viewing low-calorie food and/or non-food items. Studies that used the food cue reactivity paradigm have shed light on the intrinsic motivational significance of high-calorie compared to low-calorie foods [8] and have also noted that in food-related disorders high-calorie foods are perceived differently [17]. However, the vast majority of research studies that have used images of high and low calorie foods have employed their own set of food images, which were collected by the same authors by consulting the internet [8], [11] or cookbooks [15], therefore making it difficult to compare and replicate studies from different laboratories [18], [19].

### The IAPS and the affective picture viewing paradigm

In emotion research, the International Affective Picture System/IAPS [7] consists of approximately 1200 digitized color pictures, “representing the variety of human experience”. The IAPS is an internationally recognized, reliable tool to prompt emotional reactions, including verbal, behavioral, and psychophysiological reactions, in the laboratory (for a review, e.g., [20]). Each IAPS picture is rated by large groups of participants using the non-verbal pictorial Self-Assessment Manikin/SAM [21], to indicate whether they felt good-bad, bored-excited, and dominated-in control when looking at each picture. Thus, each picture is identified according to its basic affective dimensions of valence, arousal, and dominance. The dimensions of valence and arousal, which are essential for the expression of emotions and explain most of the variance in affective ratings, have been related to the activation of two primary motivational systems, the appetitive and the defensive systems. Furthermore, behavioral and psychophysiological data have supported the bidimensional perspective on emotions and have identified a cascade of reactions following the presentation of emotionally pleasant/unpleasant compared to neutrally affective stimuli. In clinical populations, the presentation of emotional images from the IAPS has served as a benchmark to understand the emotional processing of pathologically significant stimuli (e.g., a snake for a snake-phobic patient [22], a cigarette for an individual addicted to smoking [23], or food for a woman with bulimia nervosa [4], [24], [25]).

In the most recent IAPS [7] there are approximately 50 food images. While IAPS food pictures have not been specifically created to investigate eating pathology and therefore are not equally distributed between high and low-calorie foods, they indicate a specific pattern in emotional SAM ratings: Food is motivationally processed as moderately pleasant and moderately activating compared to neutral images, yet less pleasant and less activating compared to the most pleasant stimuli (e.g., babies, romantic couples, erotica) [19]. Thus, for emotional images researchers have identified specific contents (e.g., erotica, mutilations, personal threats) that are the most capable of activating the two primary motivational systems [26], [27]; in contrast, for food pictures it remains unclear which specific food items are the most capable of activating the appetitive motivational system. Moreover, because foods are not among the most activating emotional stimuli [26], [27], in psychophysiological research it becomes crucial to select food pictures that can more reliably prompt psychophysiological reactions.

### Blechert's and Foroni's food picture sets

Consistent with the present proposal, Foroni and colleagues [28] and Blechert and colleagues [29] have recently developed separate sets of food and non-food images for use in human eating research (“FRIDa” and “Food.pics”, respectively). In both food picture sets, foods are presented within a simple figure-ground composition and the images are digitally edited so that the food items are displayed against a white background. Both food picture sets provide researchers with information regarding each picture's nutrients, perceptual properties, and affective ratings of valence and arousal. Similar to the present proposal, both Blechert and colleagues and Foroni and colleagues aim to foster data replication, therefore facilitating the comparison of different research studies. However, neither Blechert's nor Foroni's food picture sets have been specifically created to investigate the emotional impact of food using both food and affective images from the IAPS. Accordingly, affective ratings were not obtained using SAM's classic nonverbal pictorial scales to assess affective dimensions (visual analogue scales/VAS were used, instead) and, most importantly, food images were not affectively evaluated together with emotional and neutral images from the IAPS.

### Our proposal: the Open Library of Affective Foods/OLAF

In the present research, we created a database of original food pictures and assessed their affective ratings in a large sample of participants. Food pictures were presented intermixed with affective images from the IAPS. The database has been conceived as an instrument to investigate how food is emotionally processed, thus applying the IAPS passive picture viewing paradigm (e.g., [19]) to a new set of 96 original food images, i.e., the Open Library of Affective Foods/OLAF.

The present version of the OLAF has three main features that substantially distinguish our set of images from those of Foroni and Blechert: 1) the food pictures display food items and include non-food-related items in the background, both to accurately represent human experience and to resemble affective pictures from the IAPS; 2) the food pictures are presented interspersed with emotional pictures from the IAPS and are affectively rated according to the classic affective dimensions of valence, arousal, and dominance following the methodology described in the IAPS Tech-Report [7]; 3) here, we focused on a wide sample of adolescents (affective ratings from a large group of adults are being collected in an ongoing study). The rationale for selecting a younger age group was to examine how food is emotionally processed during ages when healthy or problematic eating patterns are still under development [30], [31].

### Additional characteristics of the Open Library of Affective Foods

**Figure 1:**
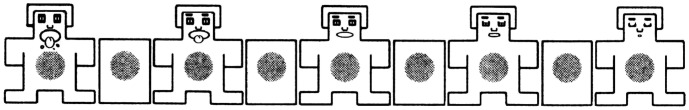
SAM food craving scale. Initially developed to assess tobacco craving [32], the SAM food craving scale ranges from a drooling to a mouth-shut SAM.

In addition to the main features previously described, some further characteristics of the OLAF set are described below. First, the food pictures are original and were obtained from real restaurants and homemade meals. We chose not to obtain images from the internet (as also in Blechert's and Foroni's picture sets) or from cookbooks [15] to increase the ecological value of the stimuli and ensure that the foods were represented in a realistic fashion. Second, the selected food images do not aim to represent the entire spectrum of foods, but focus on the extremes of a low-calorie/high-calorie food axis, therefore the final set does not include, for example, dairies, fish, and rice. The rationale here is twofold: to focus on the type of stimuli which are more commonly used to investigate the psychophysiology of eating pathology (e.g., [2], [3], [12], [16], as well as to include food stimuli which are perceptually easier to identify as more or less “appropriate for health”. The final set includes 96 pictures. Half of the pictures depict low-calorie foods, and the other half depicts high-calorie foods; these foods are further divided into fruit, vegetables, sweet high-fat foods and salty high-fat foods. Third, a new “food craving” SAM scale has been added as an extension to the three classic SAM scales of valence, arousal, and dominance [21]. This additional non-verbal pictorial scale was first developed by our research group in the context of drug addiction research [32]. It ranges from a face with a drooling mouth to a face with a mouth shut (Fig. 1). It has been included in the present study given the relevance of “food craving/desire to eat” in the perception of food stimuli [33], [34].

### Hypotheses to investigate the reliability of the OLAF

Given this background, in the present paper we test the reliability of the OLAF, which is a database of original food pictures that belong to four food categories (vegetables, fruit, sweet high-fat, and salty high-fat foods) developed to provide researchers with a standardized tool to investigate the emotional and motivational significance of food. In addition to providing the ratings of the food pictures in a large sample of adolescents, the present study also compared the ratings of the four categories of food pictures among themselves and with a subset of 36 IAPS pictures selected to represent three categories of the valence-arousal space (pleasant-high arousal, unpleasant-high arousal, and neutral-low arousal).

The replication of previous findings on both emotional and food cue processing would confirm the methodological and theoretical reliability of the OLAF database [18], [35]. Accordingly, based on previous emotion research with IAPS pictures, we predicted that: 1) similar to North American children and adolescents [36], non-food IAPS pictures in a wide sample of Spanish adolescents would prompt the expected patterns in valence, arousal, and dominance: for valence, a linear trend, with pleasant images prompting the highest valence ratings, followed by neutral and unpleasant images; for arousal, a quadratic trend, with emotional images, regardless of the valence, prompting higher arousal ratings compared to neutral images; and for dominance, a high correlation with valence ratings, with unpleasant images making participants feel the less dominant; and 2) all food pictures would be perceived as moderately pleasant and activating [19], [26]. Similarly, based on previous research on food cue processing, we predicted that: 1) high-calorie food, in our case salty and sweet high-fat, would be perceived as motivationally more relevant compared to low-calorie food [8]; and 2) that sweet high-fat food, which is a typically preferred food category [5], would receive the highest affective and food craving ratings.

## Materials and Methods

### Participants

Data from 612 participants were collected from three public high schools in downtown Granada, Spain, between October 2012 and January 2013. Affective ratings were not included for 53 adolescents (8.66%) who 1) did not rate the pictures in any of the four affective scales, 2) rated all pictures with the same value in any of the four affective scales, 3) withdrew from the study, or 4) exceeded the age limit of 17 years 3 months. Our final sample comprised self-report data from 559 adolescents (275 males and 284 females) aged 11 years 1 month through 17 years 3 months (mean 14.2 years, SD 1.4). Thus our sample, based on a categorization proposed by WHO [37], included “early” adolescence (10–14 years) and “middle” adolescence (14–17 years).

### Ethics Statement

The University of Granada Institutional Review Board approved the study and its informed consents (IRB# 699), which guaranteed the participants' confidentiality and anonymity. All participants provided written assent while their parents provided signed informed consent.

## Materials

### Affective pictures

To select affective pictures from the IAPS we referred to previous studies using the IAPS in an adolescent population [7], [36], [38]. For ethical reasons, no pictures of mutilations and erotica were included. Each affective valence category (pleasant, neutral, and unpleasant) included 12 images, which were selected to be as perceptually comparable as possible across the different affective categories. Following is the list of the 36 IAPS pictures. Pleasant: 8490, 8185, 8496, 8370, 8499, 5621, 1710, 1722, 2045, 2071, 2075, 2347; neutral: 2038, 2393, 2411, 2570, 2580, 2273, 2036, 2308, 2382, 2390, 2579, 2745.1; unpleasant: 2095, 2703, 2800, 2900, 9530, 9520, 2683, 9163, 9250, 9254, 9421, 9423. The means (and standard deviations) of the normative values in the valence scale for the selected IAPS pictures were: 7.73 (1.43) for pleasant pictures, 5.32 (1.26) for neutral pictures, and 2.29 (1.44) for unpleasant pictures. In the arousal scale the mean values were: 6.08 (2.20) for pleasant pictures, 3.27 (1.92) for neutral pictures, and 5.69 (2.18) for unpleasant pictures.

### Food images

The different food categories were identified after consulting with members of the Department of Nutrition and Bromatology from the University of Granada. Food categories were identified on two extremes: on one extreme, food whose recommended frequency of consumption is high (low-calorie foods), with the further distinction between fruits and vegetables; on the other extreme, food whose recommended frequency of consumption is low (high-calorie foods), with the further distinction between sweet high-fat and salty high-fat foods. To select food images, using a Nikon D3000 digital camera a large amount of digital pictures (approximately 2000) was first taken to depict the chosen food categories: salty high-fat, sweet high-fat, veggies, and fruits. From this initial set, up to 50 pictures were selected for each food category. Subsequently, eighteen independent raters evaluated each food picture using a 1–9 Likert scale to report the extent to which each food picture was attractive and appetizing. The ratings were subsequently converted into Z scores to circumvent individual differences in the use of the scale. The final set of 96 food pictures includes 24 images per category consistently rated as more attractive/appetizing compared with the average (all Z score ratings> = 0). Furthermore, within each food category (salty high-fat, sweet high-fat, veggies, and fruits) we identified 6 sub-types of food that were consistently rated as more appetizing (ice-cream, donuts, pastries, candies, crepes, and waffles for the sweet high-fat category; chips with egg/cheese, pizza, dumplings, tortilla/croquettes, ham, and meat dish for the salty high-fat category; fruit salad, strawberries, cut pineapple, fruit skewers, fruit slices cut, and extended fruit for the fruits category; and tomato salad, mixed salad, avocado salad, cold vegetable soup, grilled vegetables, and vegetable skewers for the veggies category). Accordingly, we included 4 different examples of each sub-type within each category. The rationale was to provide perceptually similar stimuli for future psychophysiological studies where, for example, it is necessary to create different sets of food images, while minimizing perceptual differences among sets.

### Assessment task

As it is customary in the IAPS normative ratings procedure [7], the assessment task included only 60 pictures, to avoid fatigue, and it comprised the 36 IAPS (always the same) and 24 food pictures (6 pictures per category, each one belonging to a different sub-type of food within the category). To complete the assessment of the 96 food pictures, four different pseudo-randomized picture presentation orders were prepared, each order including a different set of 24 food pictures. To control for order effects, no repetition was allowed, either of the same food category or of the same valence. Each picture presentation order began with an image from a different category (pleasant, unpleasant, food, and neutral).

### Self-report data

Food and IAPS pictures were affectively evaluated using the pictorial nonverbal SAM scales of valence, arousal, and dominance (Self-Assessment Manikin, [21]), which have been effectively used in children and adolescent populations [36]. In addition, as discussed earlier, we employed a pictorial nonverbal food “craving” SAM scale, which was previously developed by our research group in the context of drug addiction [32]. Following picture viewing, additional self-report questionnaires were collected and each participant's height and weight were assessed. Selected questionnaires investigated behaviors and personality traits associated with eating and weight-related disorders. Given the focus of the present paper on the OLAF instrument, the data on the relationships between the affective SAM ratings and the participants' characteristics will be reported elsewhere. In Table 1 we present descriptive statistics for the participants' basic characteristics (age, BMI, hunger) and personality traits (Food Craving-Trait [39], Self-Esteem, using a Spanish adaptation of Rosenberg's scale [40], [41], Sensitivity to Punishment/Sensitivity to Reward [42]).

**Table 1:** Participants characteristics.

Total Sample N559	Boys		Girls	
	N	mean (SD)	N	mean (SD)
Age (years)	275	14.37 (1.41)	284	14.15 (1.45)
BMI (kg/m2)	275	21.54 (4.19)	284	21.62 (4.15)
Hunger (y/n, 1–9) no hunger	208	2.05 (1.28)	239	1.85 (1.30)
yes, hunger	67	5.88 (2.21)	45	5.71 (2.13)
Food Craving-Trait	275	86.42 (30.99)	283	85.15 (29.53)
Self-Esteem	274	31.22 (4.79)	284	28.64 (5.53)
Sensitivity to Reward	275	8.09 (2.72)	284	6.67 (2.96)
Sensitivity to Punishment	275	5.39 (3.12)	284	7.07 (3.54)

Descriptive statistics (N and mean (standard deviation)) for some basic characteristics of the sample (age, BMI, hunger) and for personality traits (Food Craving-Trait, [39]; Self-Esteem, using a Spanish adaptation of Rosenberg's scale [40], [41]; Sensitivity to Reward/Sensitivity to Punishment, [42]). Self-reported hunger was assessed before the rating procedure began, as both a dichotomous “yes/no” variable (“Are you hungry right now?”) and as a continuous variable, using a 1–9 Likert scale (“On a 1 to 9 scale, where 1 means ‘no hunger at all’ and 9 means ‘a lot of hunger’, how much hunger do you feel right now?”).

Descriptive statistics are provided separately for boys and girls.

### Description of the Supplementary Material

In the S1 Material we provide, in three separate tables, the normative affective and food craving ratings for the 96 OLAF images. The tables display normative ratings for all participants collapsed and separately for males and females. In each table, for each OLAF picture we report: A one-word description of the displayed food item; a picture code (e.g., “fat_0012”: a code to identify each food category, “sug”, “fat”, “fru”, and “veg”, followed by four random digits); average and standard deviation ratings for valence, arousal, dominance, and food craving. Each food picture from the OLAF has been affectively evaluated, on average, by 139.75 participants (SD 6.55).

### Description of the OLAF Database

The OLAF is publicly available at https://zenodo.org with doi 10.5281/zenodo.10202 and it includes: The OLAF pictures, separated by food category and picture presentation order; the OLAF Tech Report, which includes instructions (both in Spanish and in English) and normative ratings for all participants and separately for males and females; the OLAF data files with normative ratings for all participants and separately for males and females; the OLAF raw data (each participant's code, gender, picture presentation order, IAPS and OLAF jpg picture code, experimental trial number, affective and food picture category, followed by valence, arousal, dominance, and food craving ratings for each picture).

## Procedure

The study was described to the participants and their parents as a research study on food and physical activity habits in adolescents from Southern Spain. The data were collected during school hours at assembly halls, where the participants were seated slightly apart from one another in small groups (no more than 28 individuals) and provided booklets with the SAM scales and questionnaires to fill out. Prior to the picture presentation, the participants were provided instructions on the use of the SAM scales using a Spanish translation of the IAPS instructions for children and adolescents [7], whereas the instructions for the “food craving” scale were adapted to the format of the classic SAM scales. Four practice trials, each belonging to a different category (pleasant, unpleasant, sweet high-fat food, and neutral), were added at the beginning to ensure that the participants did not have any doubt in the use of the SAM scales (pleasant: IAPS No. 2340, neutral: IAPS No. 7950, unpleasant: IAPS No. 9908, and an original picture of a birthday cake, not included in the OLAF, as a sweet high-fat food). The instructions and picture delivery were controlled using a Presentation program (v.16.3, Neurobehavioral Systems, San Francisco, CA), which was run on a Toshiba Satellite ProA120 laptop. Using an Epson EMP-54 projector, the pictures were displayed on a white screen so that the stimuli had on average a size of 2.24 m (horizontal) and 1.7 m (vertical). The screen was set at an average distance of 6.58 m from the participants, thus, on average, subtending a visual angle (19.32° horizontal and 14.72° vertical) that should maximize affective reactions to pictures [43]. The structure of each trial was as follows: first, during a 4 s inter-trial interval, the participants were informed that the next picture was about to appear, and each picture was then displayed for 6 s (the participants were invited to look at it the entire time that it was on the screen), subsequently, the participants had 20 s to rate how they felt while watching the picture using all four affective scales. As a whole, the picture presentation lasted 30 minutes. After a small break (no longer than 10 minutes and during which the participants were allowed to stand up and/or go to the restroom), the participants filled out the remaining questionnaires. Immediately prior to leaving the conference hall, each participant's height and weight were measured to estimate his/her body mass index.

## Statistical Analyses

All statistical analyses were performed using Statistica software v.8.0 (Statsoft Inc., Tulsa, USA [44]). To examine the emotional processing of the IAPS and food images, we ran separate mixed design ANOVAs on each affective scale (valence, arousal, dominance, and “food craving”) with the participants' sex as the between-subjects factor and the picture category (7 levels: pleasant, neutral, unpleasant, salty high-fat, sweet high-fat, fruit, and vegetables) as the within-subjects factor. Age, which was initially included in the analyses as a 3 level between-subjects factor (11.1–13.11, 14–15.5, 15.6–17.3 years old), was later excluded because of a lack of significance. The Greenhouse-Geisser ε correction was applied to the between-subjects factors to control for the violation of sphericity assumption. Subsequent post hoc contrasts were performed using Bonferroni-Holm's procedure [45] to adjust for multiple comparisons. The level of significance was set at p<.05 for all analyses.

## Results

### Affective Space for OLAF and IAPS images

**Figure 2:**
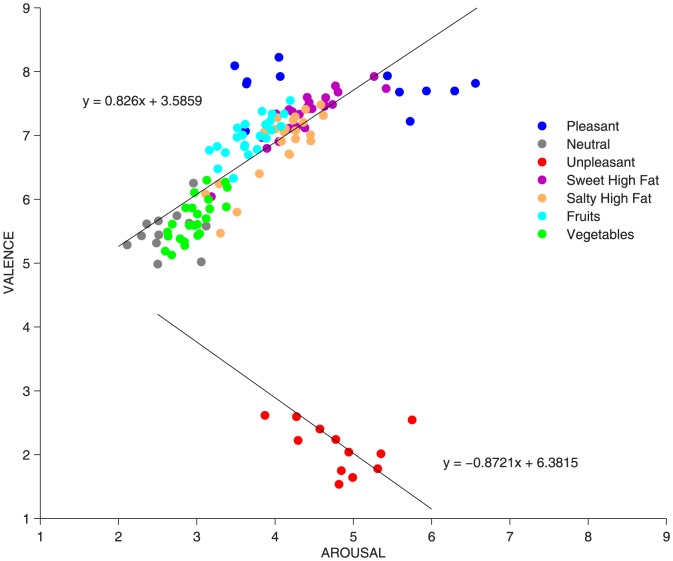
Affective Space. Bidimensional plot of each affective or food image as a function of its mean valence and arousal ratings. Each point in the plot represents the valence and arousal ratings for an IAPS (red, gray, and blue) or food picture (plotted in different colors, based on food category). The position within the “affective space” of all food and IAPS pictures display the “boomerang” shape repeatedly reported in emotion research. Regression lines are plotted separately for appetitive (valence ratings>5) and defensive contents (valence ratings <5). All food and pleasant images are located on the upper arm of the boomerang, assumed to reflect the appetitive motivational system, while fewer unpleasant images are located in the lower arm of the boomerang, corresponding to the defensive motivational system. Among the food pictures, it can be appreciated that pictures depicting vegetables lie closer to neutral contents while pictures depicting sweet high-fat foods are located closer to pleasant IAPS images.

Fig. 2 shows the two-dimensional affective space described by plotting each image according to its valence and arousal ratings, which is a standard procedure in emotion research using the IAPS [26]. Consistent with previous results also observed in young populations [36], we found that affective ratings of valence and arousal were linearly independent (r = .096, p>.05). As shown in Fig. 2, pleasant pictures occupy the upper “appetitive” arm of the space and unpleasant pictures occupy the lower “defensive” arm; both types of pictures are separated from a neutral “unemotional” area on the left. As expected, all food pictures were situated in the upper appetitive arm, which indicates the foods were processed as moderately pleasant and arousing stimuli. Among the food pictures, the sweet high-fat foods were the most capable of activating the appetitive system, as indicated by higher ratings in both valence and arousal.

### Affective space for food and emotional images from the IAPS

To further test the reliability of the ratings, we compared the affective space for affective and OLAF food images, as rated by Spanish adolescents, with the affective space for the same affective images along with all food images from the IAPS, as rated by American university students. Following is the complete list of IAPS food pictures (rated by American university students): 7200, 7220, 7230, 7250, 7255, 7260, 7270, 7279, 7280, 7281, 7282, 7283, 7284, 7285, 7286, 7287, 7289, 7290, 7291, 7300, 7320, 7325, 7330, 7340, 7350, 7351, 7352, 7354, 7365, 7390, 7400, 7402, 7405, 7410, 7430, 7440, 7450, 7451, 7460, 7461, 7470, 7472, 7475, 7476, 7477, 7480, 7481, 7482, 7484, 7487, 7488. In the last version of the IAPS [7] there are 51 food pictures, all prompting moderate activation of the appetitive motivational system, as indicated by intermediate levels of both pleasure and arousal. Moreover, 15 salty and 18 sweet high-calorie IAPS foods allowed more precise comparisons with OLAF food ratings: 1) among the food pictures, sweet high-calorie images from both sets caused the highest pleasure ratings; 2) some sweet high-calorie images from both sets reached levels of pleasure as high as some of the most pleasant non-food IAPS images included in the set, albeit being less arousing in comparison with the most pleasant non-food IAPS images.

### Valence Ratings

**Figure 3:**
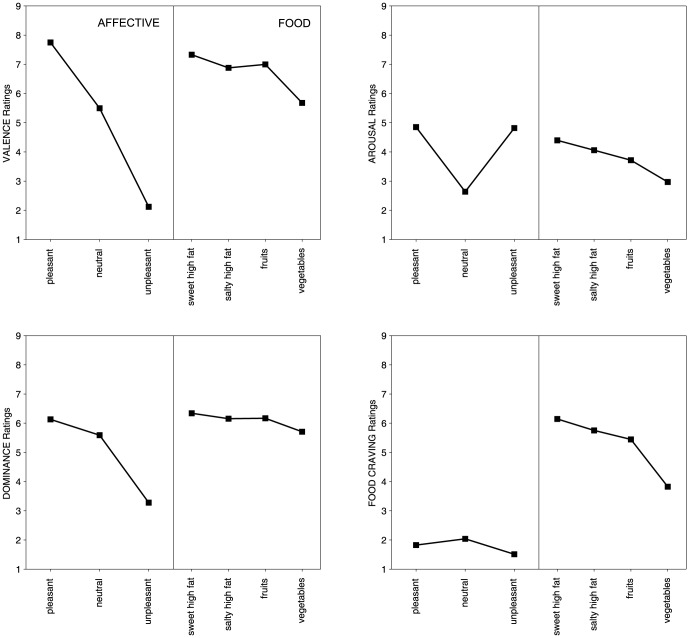
Valence, Arousal, Dominance, and Food Craving ratings across picture categories. Evaluative judgments of valence (panel a, top left), arousal (panel b, top right), dominance (panel c, bottom left), and food craving (panel d, bottom right) for affective and food picture categories. In each panel, affective categories (with pictures taken from the International Affective Picture System) are on the left, while food categories from the OLAF are on the right.

The main effect of the picture category (F(6, 3336) = 1899.7, p<.0001, partial η^2^ = 0.77), which is shown in Fig. 3a, indicated the expected decrease in valence ratings going from pleasant to unpleasant pictures (all Holm corrected ps<.0001, except for salty high-fat and fruit images, which were perceived as equally pleasant). All food pictures were perceived as moderately pleasant (pleasant>each food category>neutral); however, the pictures that depicted sweet high-fat food were the most appealing (sweet high-fat>high-fat, fruit, and veggies). A significant interaction between picture category and gender (F(6,3336) = 20.5, p<.0001, partial η^2^ = 0.03) was only the result of the IAPS affective pictures and indicated that women reacted more extremely to emotional images, feeling better than men when they looked at pleasant contents and worse than men when they looked at unpleasant contents (for pleasant pictures: women>men; for unpleasant pictures: women<men).

### Arousal Ratings

Confirming the expected pattern, the significant main effect of picture category (F(6,3336) = 232.8, p<.0001, partial η^2^ = 0.29), which is shown in Fig. 3b, indicated that emotional images, regardless of the valence, were more arousing compared to food and neutral images (pleasant  =  unpleasant; emotional images> all foods>neutrals, all ps<.0001 using Holm's post hoc). Among the food images, the participants felt progressively more excited looking at veggies, fruit, salty high-fat, and sweet high-fat foods, which indicates that sweet-high fat food produced the greatest activation in the participants. A significant interaction between gender and picture category (F(6,3336) = 3.05, p<.05, partial η^2^ = 0.005) indicated that when looking at the images of salty high-fat foods, women reported lower arousal ratings compared to men (p = .04); however, this comparison was not significant when Holm's post hoc was applied.

### Dominance Ratings

A main effect of picture category (F(6,3336) = 503.9, p<.0001, partial η^2^ = 0.47), which is shown in Fig. 3c, revealed that the participants felt the more dominant, i.e., in control, when looking at the images that depicted sweet high-fat compared to pleasant, salty high-fat, and fruit contents (these three categories made the participants feel equally dominant). Furthermore, adolescents felt less in control looking at pictures of veggies or of neutral content, whereas, as expected, they felt the less dominant when looking at unpleasant pictures (using Holm's post hoc: sweet high-fat>pleasant = fat = fruit>veggies = neutral>unpleasant). Similar to the findings emerged with the valence ratings, an additional significant interaction between gender and picture category (F(6,3336) = 17.6, p<.0001, partial η^2^ = 0.03) occurred only because of emotional images, in this case, only unpleasant images, which made female adolescents feel less in control compared to males.

### Food craving Ratings

A main effect of picture category (F(6,3336) = 1105.6, p<.0001, partial η^2^ = 0.66) revealed that the pictures that depicted sweet high-fat foods prompted the most food craving, followed by salty high-fat, fruit, and veggies (all Holm's corrected ps<.0001). As expected, neutral, pleasant, and unpleasant non-food IAPS pictures prompted the minimum food craving (see Fig. 3d). A significant main effect of gender (F(1,556) = 11.8, p<.01, partial η^2^ = 0.02) indicated that female adolescents overall reported less food craving compared to males. Additional correlational analyses on food pictures noted that food craving ratings highly correlated with valence (r = 0.96; p<.0001) and arousal (r = 0.94; p<.0001).

### Hunger and SAM ratings

The impact of hunger on affective and food craving ratings was first assessed with a mixed-design ANCoVA, which included the self-reported 1 to 9 level of hunger as a covariate and gender as a between variable. In all scales, the same main effects and interactions were observed and the overall patterns for valence, arousal, dominance, and food craving were not altered by the inclusion of hunger as a covariate. An additional statistical analysis, which included hunger as a dichotomous “yes/no” variable, indicated that participants who were hungry reported feeling stronger emotional reactions in the dimensions of valence, arousal, and food craving as compared to participants who were not hungry, as revealed by a significant main effect of hunger for valence, arousal, and food craving.

### Qualitative analysis on the most emotionally engaging and craved food items

In Table 2, we report the first ten OLAF food images, sorted by both arousal and valence, so that the first food items received the highest ratings in both valence and arousal. Among the first 10 most emotionally engaging foods (highest ratings in both pleasure and arousal), the first 9 images depicted sweet high-fat foods, whereas the 10th food picture depicted a salty high-fat meal (pizza). Moreover, among these top 10 food images, some specific food items were more represented compares to other items: waffles (4/10), pancakes (2/10), and donuts (2/10). In Table 3, we report the first ten OLAF food images based on food craving ratings. Among the top 10 most wanted foods (with the highest food craving ratings), the first 8 pictures depicted sweet high-fat foods, whereas only 2 food pictures, in the 8th and 9th places, depicted salty high-fat foods (pizza and a fast food meal). Again, among these 10 most craved foods, some food items were more represented compared to other items: waffles (3/10), pancakes (2/10), donuts (2/10). In addition, it appears noteworthy that among the top 10 most pleasant/arousing and most wanted food items, 7 of the most emotionally engaging food pictures and 7 of the most craved food pictures included chocolate as one of the main ingredients.

**Table 2:** Top ranked food items, based on valence and arousal ratings.

OLAF code	description	valence	arousal	dominance	craving	number particip	valAro ranking	craving ranking
sug_4421	Waffles	7.92	5.27	6.57	7.15	134	**1**	1
sug_0083	Candies	7.74	5.42	6.62	6.73	134	**2**	3
sug_0141	Crepes	7.77	4.77	6.59	6.68	134	**3**	5
sug_0152	Donuts	7.68	4.81	6.69	6.57	134	**4**	7
sug_0157	Donuts	7.59	4.65	6.56	6.90	135	**5**	2
sug_0014	Waffles	7.49	4.73	6.45	6.46	148	**6**	12
sug_0018	Waffles	7.49	4.63	6.42	6.72	135	**7**	4
sug_0013	Crepes	7.46	4.63	6.42	6.51	135	**8**	10
fat_5557	Pizza	7.48	4.59	6.57	6.55	148	**9**	9
sug_0113	IceCream	7.52	4.44	6.33	6.41	135	**10**	14

**Table 3:** Top ranked food items, based on food craving ratings.

OLAF code	description	valence	arousal	dominance	craving	number particip	valAro ranking	craving ranking
sug_4421	Waffles	7.92	5.27	6.57	7.15	134	1	**1**
sug_0157	Donuts	7.59	4.65	6.56	6.90	135	5	**2**
sug_0083	Candies	7.74	5.42	6.62	6.73	134	2	**3**
sug_0018	Waffles	7.49	4.63	6.42	6.72	135	7	**4**
sug_0141	Crepes	7.77	4.77	6.59	6.68	134	3	**5**
sug_0072	Waffles	7.42	4.43	6.49	6.61	142	14	**6**
sug_0152	Donuts	7.68	4.81	6.69	6.57	134	4	**7**
fat_0075	MeatDish	7.29	4.26	6.34	6.55	134	22	**8**
fat_5557	Pizza	7.48	4.59	6.57	6.55	148	9	**9**
sug_0013	Crepes	7.46	4.63	6.42	6.51	135	8	**10**

First 10 top-ranked food items, with food pictures sorted as a function of both valence and arousal ratings (Table 2) and of food craving ratings (Table 3).

Each table includes, for each food picture: its OLAF code, a description of its content, mean SAM ratings (for Valence, Arousal, Dominance, and Food Craving), the number of participants contributing to the average ratings, the ranking of that specific picture based on both valence and arousal ratings, and on food craving ratings.

## Discussion

### Reliability of the OLAF ratings

The data observed in our study using the classic SAM dimensions of valence, arousal, and dominance, were consistent with previous literature on emotion processing [26], [36] and with our hypotheses: in our sample of Spanish adolescents, affective pictures from the IAPS prompted the expected patterns in valence and arousal (linear for valence, quadratic for arousal), and dominance ratings showed the expected high correlation with valence ratings. Moreover, consistent with emotion studies [26], [27], all food categories were perceived as moderately pleasant and arousing compared to neutral images. When we examined the impact of specific food categories on affective and food craving ratings, the data that emerged were consistent with research on food cue processing [5], [8], which indicated that affective ratings were consistently higher for high-calorie food, namely salty and sweet high-fat food, and that sweet high-fat food was repeatedly the most capable of prompting appetitive emotions among food categories, as evidenced by higher ratings in valence, arousal, dominance, and craving.

As a whole, affective and food craving SAM ratings, which replicated previous literature on emotion and food cue processing, supported the methodological and theoretical reliability of the ratings included in our set of food images [35], therefore providing researchers with an effectual tool to select food images as a function of the emotions they prompt in a large sample of adolescents.

### Affective space for OLAF and IAPS food images

The position in the affective space of the food images from the IAPS (rated by American university students) and from the OLAF (rated by Spanish adolescents) indicated that: 1) sweet high-calorie images from both sets were associated with higher pleasure ratings compared to salty high-calorie images; 2) some sweet high-calorie images from both sets prompted levels of pleasure as high as some of the most pleasant non-food IAPS images included in the set, although the same food images did not reach levels of arousal as high as those prompted by pleasant non-food images. The latter observation is relevant because since arousal ratings (rather than valence) are more reliably associated with greater psychophysiological activation (e.g., [27], [46], researchers attempting to use food images to prompt reliable physiological reactions can search for food images from both sets with the highest ratings also in the arousal dimension. However, if we consider that OLAF ratings were collected in adolescents whereas IAPS ratings were collected in university students, a more direct comparison between IAPS and OLAF ratings will probably be more suitable when OLAF adult data will also be collected.

### Contribution of the OLAF

Recent psychophysiological studies [3], [4], [16], presenting IAPS and food images to healthy individuals and populations affected by eating and weight-related disorders, manifest the growing interest in investigating the emotional impact of food using physiological measures. In this context, the rationale for using both food and emotional pictures is to investigate whether altered affective responses in non-healthy populations are confined to food cues or extended to appetitive or emotional cues in general. The OLAF database aims to contribute to this line of research, therefore fostering the use of food images together with affective pictures from the IAPS. For this purpose, food pictures in our database display food items within a realistic background, matching more closely affective images from the IAPS and therefore making our database especially suitable during the recording of physiological measures (such as ERPs and fMRI) influenced by low-level features of the stimuli (e.g., [47]). In emotion research, SAM ratings associated with each IAPS picture have been crucial in facilitating reconstruction of the complex cascade of subjective, behavioral, and physiological reactions that follow the presentation of emotional and neutral stimuli of diverse content (e.g., [19]). Similarly, the OLAF database aims to identify, through language, the subjective emotional impact of each food image, with the ultimate goal of using affective ratings as an anchor to identify associated behavioral and physiological patterns.

### The SAM scales of food craving and dominance

The observed data indicated that the SAM craving scale, which was developed within our research group in the context of drug addictions [32], could reliably be applied to food items. The craving scale, designed to resemble the pictorial representation of the SAM's classic affective scales, confirmed the distribution of food items as a function of the motivational engagement defined by the remaining affective scales. The data indicated that the participants felt the greatest food craving when they looked at sweet high-fat food, which also made the participants feel the greatest pleasure. From a theoretical point of view, the dimensions of craving and pleasure refer to the distinction between ‘wanting’ and ‘liking’, which have been proposed as the basic brain reward mechanisms involved during the processing of food stimuli (e.g., [48]). Within this framework, the simultaneous examination of valence and craving ratings might illuminate the specific impact of each image on ‘liking’ and ‘wanting’ dimensions. However, when we analyzed food pictures separately, craving ratings were highly correlated with valence and arousal ratings, which suggests either that when examining the emotional impact of food, the dimensions of valence and arousal might be sufficient or that subjective ratings alone cannot disentangle the contribution of ‘wanting’ and ‘liking’ mechanisms because, according to Berridge [49], “‘liking’ and ‘wanting’ normally go together, but they can be split apart under certain circumstances, especially by certain brain manipulations”.

In emotion research, the affective dimension of dominance is typically regarded as less informative compared to the classic dimensions of valence and arousal [19], and this conception is based on the consistently high correlation between dominance and valence ratings: pleasant stimuli, including food [26], make healthy participants feel good (high valence) and in control (high dominance), whereas unpleasant stimuli make them feel bad (low valence) and out of control (low dominance). However, data from our research group repeatedly noted [24], [50] that dominance becomes highly informative in clinical populations: when food stimuli or one's own body are presented to subclinical (high food craving and/or high body dissatisfaction) or clinical (bulimia nervosa) populations, these groups feel less ‘in control’, worse, and more activated in comparison with healthy controls, hinting that the dimension of dominance, included in the OLAF adolescents ratings, could be valuable in future studies on the development of problematic eating patterns.

### Limitations and future directions

Although the data observed were highly consistent with our hypotheses, we are aware of some limitations in the present work.

First, the selected sample, which included adolescents, who ranged in age from 11 to 17 years, does not allow us to generalize observed results to an adult sample, where different food pictures might prompt the highest affective and food craving ratings. Indeed, for ethical reasons related to the youth of the sample, we did not include the IAPS affective pictures that depict mutilations, personal threats or erotica, which typically have the strongest impact on affective ratings [26]. As a result, despite our attempt to include the most arousing IAPS pictures (e.g., for pleasant pictures, adventure images; for unpleasant images, crying kids and war scenes), the absence of the most arousing affective contents had two effects on our ratings: 1) Compared with ratings previously collected in adults and children [36], we observed low arousal ratings for pleasant and unpleasant pictures, because the emotional images we selected were per se less activating than those used in earlier studies. 2) Since we did not include erotic images, gender had a different impact on pleasant picture ratings: typically, female participants rated erotic images as less pleasant compared to males [51], whereas in our case, female participants provided higher ratings for pleasant images compared to males. As a result of these considerations, we are currently collecting affective ratings from a wide sample of young adults and have included more arousing emotional IAPS images to further investigate and extend the data we observed in adolescents.

Second, although we strived to create a set of aesthetically pleasing and appetizing food images [52], we cannot a priori assume that all food images from our set will be equally likable in a non-Spanish population. However, more than a limitation in our picture set, the preference for familiar food is a characteristic of how food is processed since childhood [53]. In this context, it is evolutionarily wise that some foods are perceived as more appealing based on local availability and preference, whereas this is not the case for emotional stimuli (threatening, erotic, disgusting) which tend to be perceived similarly and independent from the culture of the viewer [54]–[56]. In our sample of Spanish adolescents, the first ten most likable, activating, and craved foods tended to be more globalized foods (such as waffles with chocolate topping, donuts or pizzas), which suggests that local food preferences had a reduced impact on preference ratings, presumably as a result of the current globalized food system.

A third limitation in the present study is that affective ratings of food and emotional pictures might also have been affected by individual characteristics (e.g., adolescents' body mass index or involvement in more or less healthy eating behaviors). This type of data, although partly recorded in the context of the present study, was not reported here because we regard these individual characteristics as not tightly related to the methodological purpose of the present investigation.

### Conclusions

The rationale behind the creation of the present set of food images is to help researchers select food images that are capable of activating the appetitive motivational system. Food images were created to perceptually match emotional pictures from the IAPS, with the long-term goal of examining, within the dimensional perspective on emotions [20], the subjective, behavioral and physiological reactions to food and affective pictures from the IAPS set.

The data reported here confirm the reliability of the affective ratings of the food pictures that form the OLAF, which is an instrument that aims to foster data replication and contributes to understanding how food is motivationally processed in healthy individuals and in patients affected by eating and weight-related disorders.

## Supporting Information

S1 Material **The normative affective and food craving ratings.** The OLAF database, publicly available at zenodo.org, includes the OLAF food images, the OLAF Tech Report, data files with normative affective and food craving ratings, and raw data files. The contents of the S1 Material and of the OLAF database are described in more detail in the Methods section of the manuscript.
(DOCX)
