# Supplementary material for: Meet OLAF, a Good Friend of the IAPS! The Open Library of Affective Foods: A Tool to Investigate the Emotional Impact of Food in Adolescents
Source: PLoS One. 2014 Dec 9;9(12):e114515. doi: 10.1371/journal.pone.0114515 (PMC4260831; doi:10.1371/journal.pone.0114515)
Supplement: S1 Material — The normative affective and food craving ratings. The OLAF database, publicly available at zenodo.org, includes the OLAF food images, the OLAF Tech Report, data files with normative affective and food craving ratings, and raw data files. The contents of the S1 Material and of the OLAF database are described in more detail in the Methods section of the manuscript. (DOCX) [file pone.0114515.s001.docx]

| Description | Pic  Code | Valence  Mean (SD) | Arousal  Mean (SD) | Dominance  Mean (SD) | Craving  Mean (SD) |
| --- | --- | --- | --- | --- | --- |
|  |  |  |  |  |  |
| ChipsCheese | fat_0012 | 6,24(2,14) | 3,28(2,47) | 6,05(2,30) | 4,82(2,99) |
| ChipsEggs | fat_0018 | 7,05(2,04) | 3,86(2,82) | 6,08(2,17) | 5,93(2,87) |
| ChipsEggs | fat_0022 | 6,71(1,79) | 4,18(2,57) | 6,02(1,82) | 5,67(2,75) |
| MeatDish | fat_0029 | 6,70(1,97) | 4,19(2,67) | 5,90(2,11) | 5,91(2,81) |
| Ham | fat_0036 | 7,10(2,01) | 4,00(2,87) | 6,10(2,40) | 5,91(2,87) |
| ChipsCheese | fat_0037 | 7,24(1,96) | 4,23(2,78) | 6,40(1,98) | 6,51(2,69) |
| Tortilla | fat_0038 | 7,16(1,73) | 3,88(2,62) | 6,28(2,20) | 5,91(2,72) |
| Dumplings | fat_0042 | 5,80(2,20) | 3,52(2,63) | 5,56(1,98) | 4,01(2,92) |
| MeatDish | fat_0055 | 6,91(2,06) | 4,08(2,70) | 6,02(2,37) | 5,77(2,97) |
| MeatDish | fat_0075 | 7,29(1,76) | 4,26(2,79) | 6,34(1,90) | 6,55(2,59) |
| Croquettes | fat_0083 | 7,28(1,74) | 4,03(2,83) | 6,30(2,19) | 6,13(2,91) |
| MeatDish | fat_0107 | 6,95(2,06) | 4,26(2,76) | 6,34(2,29) | 6,12(2,85) |
| Ham | fat_0118 | 7,21(2,11) | 4,36(2,85) | 6,34(2,42) | 6,26(2,85) |
| Ham | fat_0156 | 7,10(1,88) | 4,13(2,90) | 6,10(1,97) | 6,05(2,78) |
| Ham | fat_0242 | 7,02(2,02) | 4,45(3,01) | 6,59(1,94) | 6,15(2,83) |
| Dumplings | fat_0611 | 6,08(1,97) | 3,11(2,32) | 5,90(2,06) | 4,42(2,76) |
| Pizza | fat_0655 | 7,41(2,05) | 4,39(3,05) | 6,28(2,36) | 6,45(2,89) |
| Dumplings | fat_0721 | 5,47(2,19) | 3,30(2,40) | 5,70(2,44) | 4,29(2,92) |
| Tortilla | fat_5515 | 7,06(1,67) | 4,11(2,71) | 6,25(1,86) | 5,74(2,74) |
| Pizza | fat_5557 | 7,48(1,96) | 4,59(3,00) | 6,57(2,32) | 6,55(2,87) |

| Description | Pic  Code | Valence  Mean (SD) | Arousal  Mean (SD) | Dominance  Mean (SD) | Craving  Mean (SD) |
| --- | --- | --- | --- | --- | --- |
|  |  |  |  |  |  |
| Pizza | fat_6053 | 6,40(2,21) | 3,80(2,88) | 6,00(2,10) | 4,69(3,16) |
| Pizza | fat_6054 | 7,07(1,85) | 4,26(2,76) | 6,25(1,72) | 5,81(2,85) |
| Dumplings | fat_6439 | 6,92(2,09) | 4,46(2,98) | 6,16(1,98) | 6,01(2,88) |
| Croquettes | fatfood3 | 7,31(1,86) | 4,62(2,93) | 6,34(1,98) | 6,35(2,71) |
| FruitSkewers | fru_0015 | 7,01(1,98) | 3,59(2,74) | 6,23(2,00) | 5,53(2,95) |
| FruitSalad | fru_0024 | 7,17(2,01) | 3,62(2,66) | 6,10(1,92) | 5,52(2,91) |
| FruitSlicesCut | fru_0083 | 7,12(1,61) | 3,52(2,45) | 6,23(1,89) | 5,62(2,68) |
| FruitSlicesCut | fru_0103 | 7,34(1,77) | 3,96(2,65) | 6,27(2,16) | 5,75(2,81) |
| Strawberry | fru_0144 | 7,54(1,50) | 4,20(3,03) | 6,41(1,84) | 5,95(2,81) |
| FruitSalad | fru_0190 | 6,77(1,79) | 3,16(2,27) | 6,10(1,97) | 5,30(2,78) |
| ExtendedFruit | fru_0289 | 6,95(1,71) | 3,89(2,91) | 6,25(1,90) | 5,65(2,79) |
| Strawberry | fru_0337 | 7,34(1,56) | 4,12(2,75) | 6,33(1,93) | 5,60(2,81) |
| FruitSkewers | fru_0384 | 6,83(1,86) | 3,26(2,47) | 6,14(2,23) | 5,06(2,95) |
| CutPineapple | fru_0492 | 6,97(1,73) | 3,51(2,49) | 5,93(1,77) | 5,24(2,94) |
| CutPineapple | fru_0495 | 6,99(1,78) | 3,81(2,72) | 6,32(1,91) | 5,35(2,84) |
| FruitSkewers | fru_0511 | 7,23(1,60) | 3,94(2,70) | 6,29(1,92) | 5,72(2,66) |
| FruitSlicesCut | fru_0553 | 6,33(2,04) | 3,47(2,58) | 5,85(2,17) | 4,65(2,96) |
| FruitSkewers | fru_0593 | 6,73(2,10) | 3,37(2,60) | 6,01(2,40) | 5,36(2,90) |
| ExtendedFruit | fru_0613 | 6,78(1,88) | 3,77(2,62) | 6,08(2,01) | 5,23(2,96) |
| FruitSalad | fru_0670 | 7,38(1,51) | 3,84(2,75) | 6,45(1,89) | 5,77(2,43) |

| Description | Pic  Code | Valence  Mean (SD) | Arousal  Mean (SD) | Dominance  Mean (SD) | Craving  Mean (SD) |
| --- | --- | --- | --- | --- | --- |
|  |  |  |  |  |  |
| FruitSalad | fru_0683 | 7,14(1,85) | 4,08(2,73) | 6,36(2,11) | 5,96(2,73) |
| ExtendedFruit | fru_1902 | 6,85(2,09) | 3,61(2,53) | 5,99(2,38) | 5,37(2,81) |
| CutPineapple | fru_5515 | 6,83(1,97) | 3,61(2,53) | 6,15(2,19) | 5,41(2,75) |
| FruitSlicesCut | fru_5595 | 6,70(2,00) | 3,66(2,61) | 6,10(2,28) | 5,47(2,81) |
| CutPineapple | fru_5772 | 6,48(2,11) | 3,27(2,62) | 6,00(2,11) | 4,59(2,84) |
| Strawberry | fru_5782 | 7,16(1,91) | 3,89(2,76) | 6,18(2,29) | 5,47(2,94) |
| Strawberry | fru_5795 | 7,08(1,89) | 3,95(2,80) | 6,23(2,15) | 5,54(2,82) |
| ExtendedFruit | fru_5833 | 7,17(1,90) | 3,88(2,84) | 6,20(2,18) | 5,47(2,94) |
| Crepes | sug_0013 | 7,46(1,94) | 4,63(2,76) | 6,42(2,04) | 6,51(2,62) |
| Waffles | sug_0014 | 7,49(1,85) | 4,73(2,86) | 6,45(2,21) | 6,46(2,85) |
| Waffles | sug_0018 | 7,49(1,79) | 4,63(3,01) | 6,42(2,19) | 6,72(2,62) |
| Donuts | sug_0043 | 7,33(2,10) | 4,31(2,89) | 6,04(2,33) | 6,34(2,75) |
| Waffles | sug_0072 | 7,42(2,13) | 4,43(3,00) | 6,49(2,21) | 6,61(2,76) |
| Candies | sug_0083 | 7,74(1,88) | 5,42(2,99) | 6,62(2,09) | 6,73(2,77) |
| Candies | sug_0096 | 7,42(1,90) | 4,47(2,81) | 6,30(1,99) | 6,01(2,92) |
| Candies | sug_0099 | 7,38(1,80) | 4,22(2,65) | 6,16(2,26) | 5,77(2,73) |
| Candies | sug_0101 | 7,44(1,98) | 4,42(2,98) | 6,43(2,34) | 5,99(2,70) |
| IceCream | sug_0112 | 7,34(1,79) | 4,01(2,87) | 6,31(2,32) | 5,88(2,81) |
| IceCream | sug_0113 | 7,52(1,73) | 4,44(3,00) | 6,33(2,01) | 6,41(2,70) |
| IceCream | sug_0116 | 6,04(1,99) | 3,19(2,46) | 5,93(2,33) | 4,13(2,73) |

| Description | Pic  Code | Valence  Mean (SD) | Arousal  Mean (SD) | Dominance  Mean (SD) | Craving  Mean (SD) |
| --- | --- | --- | --- | --- | --- |
|  |  |  |  |  |  |
| IceCream | sug_0135 | 7,60(1,67) | 4,41(2,83) | 6,50(2,02) | 6,30(2,76) |
| Crepes | sug_0141 | 7,77(1,45) | 4,77(2,86) | 6,59(1,97) | 6,68(2,66) |
| Pastries | sug_0147 | 6,80(2,05) | 3,90(2,80) | 5,99(2,12) | 5,68(2,88) |
| Donuts | sug_0150 | 7,41(1,81) | 4,18(2,68) | 6,54(2,00) | 6,27(2,80) |
| Pastries | sug_0151 | 6,96(2,00) | 3,84(2,83) | 6,41(2,13) | 5,46(2,95) |
| Donuts | sug_0152 | 7,68(1,58) | 4,81(2,92) | 6,69(1,80) | 6,57(2,65) |
| Donuts | sug_0157 | 7,59(1,78) | 4,65(3,04) | 6,56(2,10) | 6,90(2,46) |
| Pastries | sug_0166 | 6,90(1,95) | 4,05(2,68) | 6,13(2,13) | 5,52(2,92) |
| Pastries | sug_152. | 7,11(1,69) | 4,18(2,75) | 6,22(1,88) | 5,43(2,90) |
| Crepes | sug_4009 | 7,12(1,97) | 4,38(2,85) | 6,20(2,21) | 6,28(2,90) |
| Crepes | sug_4011 | 7,13(2,05) | 4,27(2,89) | 6,05(2,28) | 6,03(2,83) |
| Waffles | sug_4421 | 7,92(1,57) | 5,27(3,02) | 6,57(2,20) | 7,15(2,53) |
| ColdSoup | veg_0002 | 5,85(2,33) | 3,16(2,43) | 6,08(2,24) | 4,14(2,97) |
| VegetableSkewer | veg_0005 | 5,13(1,97) | 2,68(1,96) | 5,36(2,19) | 3,31(2,49) |
| TomatoSalad | veg_0011 | 5,46(2,19) | 3,04(2,47) | 5,55(2,16) | 3,62(2,63) |
| AvocadoSalad | veg_0012 | 5,59(2,00) | 2,97(2,32) | 5,56(2,02) | 3,60(2,73) |
| ColdSoup | veg_0048 | 5,19(1,93) | 2,60(2,08) | 5,15(2,45) | 3,25(2,61) |
| AvocadoSalad | veg_0070 | 5,86(1,90) | 2,86(2,35) | 5,94(1,84) | 3,58(2,84) |
| GrilledVegetables | veg_0079 | 5,34(2,19) | 2,84(2,31) | 5,51(2,15) | 3,51(2,85) |
| VegetableSkewer | veg_0085 | 5,87(1,85) | 2,94(2,29) | 5,89(1,88) | 3,53(2,62) |

| Description | Pic  Code | Valence  Mean (SD) | Arousal  Mean (SD) | Dominance  Mean (SD) | Craving  Mean (SD) |
| --- | --- | --- | --- | --- | --- |
|  |  |  |  |  |  |
| VegetableSkewer | veg_0092 | 5,38(2,13) | 2,79(2,42) | 5,63(2,15) | 3,30(2,71) |
| TomatoSalad | veg_0109 | 6,27(1,96) | 3,37(2,48) | 6,03(2,42) | 4,63(3,00) |
| ColdSoup | veg_0113 | 5,61(2,21) | 2,69(2,25) | 5,63(2,06) | 3,75(2,88) |
| TomatoSalad | veg_0114 | 6,30(1,92) | 3,13(2,44) | 5,90(1,76) | 4,42(2,80) |
| VegetableSkewer | veg_0118 | 5,43(2,37) | 3,01(2,25) | 5,49(2,44) | 3,99(2,86) |
| MixedSalad | veg_0125 | 6,19(2,05) | 3,39(2,59) | 5,99(2,31) | 4,41(3,00) |
| AvocadoSalad | veg_0133 | 5,42(1,86) | 2,63(2,13) | 5,48(2,11) | 3,06(2,47) |
| MixedSalad | veg_0135 | 6,11(1,89) | 2,97(2,53) | 5,93(1,96) | 4,06(2,75) |
| AvocadoSalad | veg_0148 | 5,49(2,06) | 2,63(1,96) | 5,53(2,41) | 3,83(2,81) |
| MixedSalad | veg_0152 | 5,61(1,98) | 3,01(2,26) | 5,63(2,10) | 3,74(2,84) |
| GrilledVegetables | veg_0186 | 5,28(2,00) | 2,84(2,22) | 5,65(2,06) | 3,45(2,64) |
| GrilledVegetables | veg_0198 | 5,88(1,99) | 3,38(2,33) | 5,72(2,30) | 4,54(2,87) |
| MixedSalad | veg_0281 | 5,59(2,00) | 2,91(2,13) | 5,91(2,18) | 3,77(2,61) |
| ColdSoup | veg_0455 | 6,00(2,06) | 3,15(2,45) | 5,94(1,91) | 4,35(3,04) |
| TomatoSalad | veg_0725 | 5,70(2,08) | 3,12(2,43) | 5,73(2,02) | 3,91(2,71) |
| GrilledVegetables | vegofood | 5,77(2,06) | 3,01(2,50) | 5,99(1,95) | 3,8(2,920) |

| Description | Pic  Code | Valence  Mean (SD) | Arousal  Mean (SD) | Dominance  Mean (SD) | Craving  Mean (SD) |
| --- | --- | --- | --- | --- | --- |
|  |  |  |  |  |  |
| ChipsCheese | fat_0012 | 6,29(2,03) | 3,33(2,51) | 5,81(2,45) | 5,20(2,89) |
| ChipsEggs | fat_0018 | 7,11(1,69) | 4,13(2,88) | 6,66(2,04) | 6,51(2,57) |
| ChipsEggs | fat_0022 | 6,72(1,86) | 4,24(2,65) | 6,21(1,71) | 5,84(2,76) |
| MeatDish | fat_0029 | 6,63(2,11) | 4,22(2,73) | 6,10(2,23) | 6,00(2,78) |
| Ham | fat_0036 | 7,03(1,93) | 4,41(3,14) | 6,50(2,51) | 6,19(2,74) |
| ChipsCheese | fat_0037 | 7,17(1,94) | 4,42(2,88) | 6,55(1,84) | 6,86(2,64) |
| Tortilla | fat_0038 | 7,50(1,62) | 4,04(2,68) | 6,27(2,24) | 6,29(2,70) |
| Dumplings | fat_0042 | 5,96(2,36) | 3,61(2,62) | 5,78(1,83) | 4,12(3,07) |
| MeatDish | fat_0055 | 7,20(1,82) | 4,58(2,72) | 6,23(2,47) | 6,35(2,55) |
| MeatDish | fat_0075 | 7,36(1,69) | 4,42(2,87) | 6,45(1,78) | 7,11(2,46) |
| Croquettes | fat_0083 | 7,34(1,63) | 4,13(2,92) | 6,50(2,16) | 6,57(2,61) |
| MeatDish | fat_0107 | 6,96(2,09) | 4,27(2,78) | 6,27(2,55) | 6,37(2,84) |
| Ham | fat_0118 | 7,51(2,00) | 4,61(2,84) | 6,51(2,38) | 6,49(2,72) |
| Ham | fat_0156 | 7,06(2,01) | 4,24(2,96) | 6,07(1,98) | 6,22(2,85) |
| Ham | fat_0242 | 6,97(1,85) | 4,46(3,09) | 6,54(2,02) | 6,55(2,73) |
| Dumplings | fat_0611 | 6,15(1,98) | 3,44(2,48) | 6,11(2,14) | 4,97(2,62) |
| Pizza | fat_0655 | 7,63(1,66) | 4,79(3,12) | 6,43(2,34) | 7,09(2,50) |
| Dumplings | fat_0721 | 5,47(2,28) | 3,41(2,37) | 5,79(2,43) | 4,93(2,89) |
| Tortilla | fat_5515 | 6,94(1,84) | 4,03(2,89) | 6,12(2,03) | 5,78(2,96) |
| Pizza | fat_5557 | 7,44(2,06) | 4,66(3,17) | 6,48(2,48) | 6,85(2,84) |

| Description | Pic  Code | Valence  Mean (SD) | Arousal  Mean (SD) | Dominance  Mean (SD) | Craving  Mean (SD) |
| --- | --- | --- | --- | --- | --- |
|  |  |  |  |  |  |
| Pizza | fat_6053 | 6,63(2,28) | 3,87(2,93) | 6,26(2,19) | 4,84(3,25) |
| Pizza | fat_6054 | 7,09(1,74) | 4,66(2,88) | 6,18(1,84) | 6,45(2,68) |
| Dumplings | fat_6439 | 7,15(1,76) | 4,92(3,01) | 6,08(2,09) | 6,69(2,52) |
| Croquettes | fatfood3 | 7,35(1,71) | 4,97(2,95) | 6,38(2,07) | 6,69(2,49) |
| FruitSkewers | fru_0015 | 6,69(2,12) | 3,31(2,45) | 6,10(2,07) | 5,50(3,03) |
| FruitSalad | fru_0024 | 7,16(2,16) | 3,39(2,65) | 6,44(1,87) | 5,47(3,10) |
| FruitSlicesCut | fru_0083 | 7,03(1,62) | 3,45(2,32) | 6,23(1,76) | 5,88(2,65) |
| FruitSlicesCut | fru_0103 | 7,04(1,95) | 3,59(2,64) | 6,22(2,03) | 5,60(3,00) |
| Strawberry | fru_0144 | 7,38(1,51) | 4,56(3,12) | 6,46(1,81) | 6,25(2,68) |
| FruitSalad | fru_0190 | 6,66(1,72) | 3,16(2,21) | 6,46(2,00) | 5,44(2,70) |
| ExtendedFruit | fru_0289 | 6,78(1,73) | 3,69(2,70) | 6,09(1,81) | 5,68(2,70) |
| Strawberry | fru_0337 | 7,19(1,61) | 3,87(2,80) | 6,31(1,95) | 5,66(2,97) |
| FruitSkewers | fru_0384 | 6,93(1,67) | 3,56(2,67) | 6,59(2,16) | 5,23(2,92) |
| CutPineapple | fru_0492 | 6,88(1,72) | 3,55(2,52) | 6,00(1,85) | 5,38(2,98) |
| CutPineapple | fru_0495 | 7,16(1,46) | 4,16(2,63) | 6,20(1,89) | 5,92(2,64) |
| FruitSkewers | fru_0511 | 7,03(1,57) | 3,83(2,66) | 6,28(1,86) | 5,72(2,55) |
| FruitSlicesCut | fru_0553 | 6,30(1,92) | 3,63(2,68) | 5,82(2,33) | 4,80(2,91) |
| FruitSkewers | fru_0593 | 6,79(2,07) | 3,49(2,63) | 6,00(2,48) | 5,83(2,87) |
| ExtendedFruit | fru_0613 | 6,68(1,97) | 3,66(2,61) | 6,06(2,12) | 5,35(3,05) |
| FruitSalad | fru_0670 | 7,09(1,51) | 3,80(2,68) | 6,32(1,82) | 5,85(2,33) |

| Description | Pic  Code | Valence  Mean (SD) | Arousal  Mean (SD) | Dominance  Mean (SD) | Craving  Mean (SD) |
| --- | --- | --- | --- | --- | --- |
|  |  |  |  |  |  |
| FruitSalad | fru_0683 | 7,17(1,81) | 4,17(2,82) | 6,48(2,20) | 6,21(2,75) |
| ExtendedFruit | fru_1902 | 6,90(1,96) | 3,53(2,61) | 6,03(2,41) | 5,90(2,68) |
| CutPineapple | fru_5515 | 6,51(2,23) | 3,43(2,44) | 5,83(2,22) | 5,46(2,87) |
| FruitSlicesCut | fru_5595 | 6,84(1,84) | 3,76(2,50) | 6,03(2,38) | 5,79(2,69) |
| CutPineapple | fru_5772 | 6,60(2,09) | 3,69(2,78) | 6,34(2,08) | 5,21(2,68) |
| Strawberry | fru_5782 | 7,14(1,78) | 3,75(2,71) | 6,07(2,34) | 5,85(2,86) |
| Strawberry | fru_5795 | 6,90(1,85) | 4,41(2,88) | 6,27(2,29) | 5,83(2,61) |
| ExtendedFruit | fru_5833 | 7,03(1,92) | 4,26(3,04) | 6,23(2,17) | 5,89(2,86) |
| Crepes | sug_0013 | 7,06(2,16) | 4,21(2,71) | 6,50(1,94) | 6,40(2,74) |
| Waffles | sug_0014 | 7,55(1,82) | 4,97(2,95) | 6,49(2,24) | 6,94(2,60) |
| Waffles | sug_0018 | 7,18(2,02) | 4,25(3,01) | 6,37(2,04) | 6,81(2,61) |
| Donuts | sug_0043 | 7,35(1,96) | 4,82(2,85) | 6,24(2,28) | 6,58(2,61) |
| Waffles | sug_0072 | 7,26(1,81) | 4,54(3,01) | 6,67(2,26) | 6,71(2,62) |
| Candies | sug_0083 | 7,54(1,80) | 5,83(2,83) | 6,51(2,05) | 6,94(2,60) |
| Candies | sug_0096 | 7,04(2,15) | 4,31(2,90) | 6,26(1,86) | 6,15(2,99) |
| Candies | sug_0099 | 7,07(1,86) | 4,06(2,44) | 6,12(2,35) | 5,72(2,63) |
| Candies | sug_0101 | 7,27(1,78) | 4,72(3,02) | 6,66(2,20) | 6,08(2,50) |
| IceCream | sug_0112 | 7,43(1,66) | 4,24(2,86) | 6,60(2,36) | 6,30(2,64) |
| IceCream | sug_0113 | 7,35(1,97) | 4,57(3,11) | 6,32(2,11) | 6,71(2,67) |
| IceCream | sug_0116 | 5,99(2,11) | 3,13(2,22) | 5,82(2,56) | 4,37(2,65) |

| Description | Pic  Code | Valence  Mean (SD) | Arousal  Mean (SD) | Dominance  Mean (SD) | Craving  Mean (SD) |
| --- | --- | --- | --- | --- | --- |
|  |  |  |  |  |  |
| IceCream | sug_0135 | 7,48(1,75) | 4,42(2,80) | 6,44(2,10) | 6,58(2,74) |
| Crepes | sug_0141 | 7,64(1,54) | 4,94(2,87) | 6,53(1,88) | 6,86(2,75) |
| Pastries | sug_0147 | 6,76(2,12) | 3,88(2,94) | 5,68(2,30) | 6,18(2,97) |
| Donuts | sug_0150 | 7,41(1,81) | 4,37(2,65) | 6,71(1,93) | 6,40(2,83) |
| Pastries | sug_0151 | 7,03(1,99) | 4,03(3,01) | 6,83(2,12) | 6,00(2,74) |
| Donuts | sug_0152 | 7,46(1,74) | 4,69(2,84) | 6,52(1,92) | 6,82(2,51) |
| Donuts | sug_0157 | 7,45(2,00) | 4,55(3,09) | 6,55(2,22) | 7,25(2,43) |
| Pastries | sug_0166 | 7,00(1,95) | 4,31(2,49) | 6,09(2,17) | 5,73(2,83) |
| Pastries | sug_152. | 7,14(1,69) | 4,62(2,85) | 6,28(1,79) | 6,09(2,83) |
| Crepes | sug_4009 | 6,96(2,16) | 4,47(2,70) | 5,87(2,34) | 6,57(2,77) |
| Crepes | sug_4011 | 7,18(1,81) | 4,55(2,95) | 6,18(2,26) | 6,06(2,62) |
| Waffles | sug_4421 | 7,58(1,73) | 5,38(3,11) | 6,34(2,35) | 7,08(2,68) |
| ColdSoup | veg_0002 | 5,87(2,40) | 3,34(2,39) | 5,90(2,27) | 4,40(2,94) |
| VegetableSkewer | veg_0005 | 5,13(2,05) | 2,94(2,15) | 5,43(2,34) | 3,69(2,46) |
| TomatoSalad | veg_0011 | 5,19(2,18) | 3,37(2,66) | 5,84(2,28) | 3,54(2,48) |
| AvocadoSalad | veg_0012 | 5,51(2,22) | 3,27(2,57) | 5,82(2,03) | 3,77(2,82) |
| ColdSoup | veg_0048 | 5,03(1,86) | 2,43(1,84) | 5,10(2,56) | 2,89(2,26) |
| AvocadoSalad | veg_0070 | 5,84(1,75) | 3,02(2,41) | 5,98(1,74) | 3,79(2,83) |
| GrilledVegetables | veg_0079 | 5,19(2,36) | 2,72(2,17) | 5,76(2,24) | 3,53(2,85) |
| VegetableSkewer | veg_0085 | 5,82(1,86) | 3,14(2,39) | 5,88(1,82) | 3,88(2,60) |

| Description | Pic  Code | Valence  Mean (SD) | Arousal  Mean (SD) | Dominance  Mean (SD) | Craving  Mean (SD) |
| --- | --- | --- | --- | --- | --- |
|  |  |  |  |  |  |
| VegetableSkewer | veg_0092 | 5,63(2,36) | 3,03(2,53) | 5,97(2,14) | 3,74(2,88) |
| TomatoSalad | veg_0109 | 6,35(2,01) | 3,62(2,58) | 5,84(2,51) | 4,83(2,98) |
| ColdSoup | veg_0113 | 5,72(2,48) | 3,01(2,56) | 5,80(2,27) | 3,87(3,08) |
| TomatoSalad | veg_0114 | 6,25(1,71) | 3,25(2,36) | 5,88(1,78) | 5,00(2,66) |
| VegetableSkewer | veg_0118 | 5,77(2,40) | 3,39(2,33) | 5,39(2,59) | 4,57(2,96) |
| MixedSalad | veg_0125 | 6,25(2,05) | 3,49(2,53) | 5,79(2,31) | 4,69(2,90) |
| AvocadoSalad | veg_0133 | 5,34(1,75) | 2,77(2,02) | 5,49(2,28) | 3,04(2,37) |
| MixedSalad | veg_0135 | 6,14(1,82) | 3,39(2,75) | 5,64(2,02) | 4,52(2,76) |
| AvocadoSalad | veg_0148 | 5,61(2,25) | 2,60(1,86) | 5,27(2,47) | 4,17(2,90) |
| MixedSalad | veg_0152 | 5,55(2,06) | 3,06(2,33) | 5,90(1,90) | 3,90(2,98) |
| GrilledVegetables | veg_0186 | 5,31(1,84) | 3,00(2,30) | 5,80(1,99) | 3,50(2,57) |
| GrilledVegetables | veg_0198 | 6,19(1,94) | 3,54(2,45) | 5,84(2,47) | 4,80(2,99) |
| MixedSalad | veg_0281 | 5,35(2,07) | 2,87(2,16) | 6,01(2,29) | 3,96(2,56) |
| ColdSoup | veg_0455 | 6,03(1,90) | 3,43(2,56) | 6,03(1,74) | 4,85(3,00) |
| TomatoSalad | veg_0725 | 5,78(2,16) | 3,29(2,54) | 6,14(1,81) | 4,03(2,71) |
| GrilledVegetables | vegofood | 5,77(2,08) | 3,29(2,60) | 6,03(1,92) | 4,29(2,90) |

| Description | Pic  Code | Valence  Mean (SD) | Arousal  Mean (SD) | Dominance  Mean (SD) | Craving  Mean (SD) |
| --- | --- | --- | --- | --- | --- |
|  |  |  |  |  |  |
| ChipsCheese | fat_0012 | 6,20(2,25) | 3,24(2,45) | 6,27(2,13) | 4,46(3,06) |
| ChipsEggs | fat_0018 | 6,99(2,34) | 3,59(2,75) | 5,51(2,16) | 5,35(3,04) |
| ChipsEggs | fat_0022 | 6,71(1,74) | 4,12(2,51) | 5,83(1,93) | 5,50(2,74) |
| MeatDish | fat_0029 | 6,77(1,84) | 4,15(2,62) | 5,69(1,97) | 5,82(2,86) |
| Ham | fat_0036 | 7,17(2,10) | 3,59(2,54) | 5,70(2,24) | 5,65(2,99) |
| ChipsCheese | fat_0037 | 7,30(1,99) | 4,06(2,69) | 6,26(2,11) | 6,19(2,72) |
| Tortilla | fat_0038 | 6,86(1,77) | 3,74(2,57) | 6,28(2,18) | 5,57(2,72) |
| Dumplings | fat_0042 | 5,63(2,02) | 3,42(2,65) | 5,33(2,12) | 3,89(2,77) |
| MeatDish | fat_0055 | 6,62(2,26) | 3,59(2,61) | 5,82(2,27) | 5,20(3,25) |
| MeatDish | fat_0075 | 7,22(1,84) | 4,10(2,71) | 6,24(2,01) | 6,01(2,61) |
| Croquettes | fat_0083 | 7,21(1,85) | 3,93(2,76) | 6,11(2,22) | 5,70(3,14) |
| MeatDish | fat_0107 | 6,93(2,04) | 4,26(2,77) | 6,41(2,02) | 5,89(2,86) |
| Ham | fat_0118 | 6,92(2,18) | 4,13(2,85) | 6,17(2,46) | 6,05(2,96) |
| Ham | fat_0156 | 7,15(1,75) | 4,03(2,84) | 6,13(1,98) | 5,88(2,72) |
| Ham | fat_0242 | 7,06(2,18) | 4,44(2,95) | 6,63(1,87) | 5,76(2,89) |
| Dumplings | fat_0611 | 6,01(1,98) | 2,79(2,11) | 5,69(1,98) | 3,86(2,79) |
| Pizza | fat_0655 | 7,20(2,36) | 4,00(2,96) | 6,13(2,38) | 5,81(3,12) |
| Dumplings | fat_0721 | 5,47(2,11) | 3,20(2,43) | 5,63(2,45) | 3,69(2,83) |
| Tortilla | fat_5515 | 7,18(1,50) | 4,19(2,54) | 6,37(1,67) | 5,70(2,51) |
| Pizza | fat_5557 | 7,52(1,87) | 4,52(2,85) | 6,65(2,18) | 6,27(2,90) |

| Description | Pic  Code | Valence  Mean (SD) | Arousal  Mean (SD) | Dominance  Mean (SD) | Craving  Mean (SD) |
| --- | --- | --- | --- | --- | --- |
|  |  |  |  |  |  |
| Pizza | fat_6053 | 6,17(2,13) | 3,74(2,85) | 5,74(1,99) | 4,54(3,08) |
| Pizza | fat_6054 | 7,06(1,97) | 3,88(2,61) | 6,32(1,60) | 5,20(2,88) |
| Dumplings | fat_6439 | 6,69(2,35) | 4,01(2,91) | 6,24(1,88) | 5,35(3,07) |
| Croquettes | fatfood3 | 7,28(2,00) | 4,29(2,89) | 6,29(1,91) | 6,03(2,87) |
| FruitSkewers | fru_0015 | 7,33(1,78) | 3,87(2,99) | 6,36(1,94) | 5,55(2,90) |
| FruitSalad | fru_0024 | 7,18(1,87) | 3,86(2,68) | 5,75(1,93) | 5,58(2,72) |
| FruitSlicesCut | fru_0083 | 7,20(1,61) | 3,58(2,59) | 6,22(2,01) | 5,39(2,70) |
| FruitSlicesCut | fru_0103 | 7,64(1,51) | 4,34(2,63) | 6,31(2,29) | 5,90(2,62) |
| Strawberry | fru_0144 | 7,70(1,50) | 3,86(2,93) | 6,36(1,89) | 5,68(2,93) |
| FruitSalad | fru_0190 | 6,87(1,86) | 3,16(2,34) | 5,75(1,89) | 5,16(2,87) |
| ExtendedFruit | fru_0289 | 7,12(1,68) | 4,07(3,11) | 6,41(1,99) | 5,62(2,90) |
| Strawberry | fru_0337 | 7,49(1,50) | 4,37(2,70) | 6,34(1,93) | 5,54(2,65) |
| FruitSkewers | fru_0384 | 6,73(2,04) | 2,97(2,25) | 5,70(2,23) | 4,90(2,99) |
| CutPineapple | fru_0492 | 7,06(1,76) | 3,48(2,47) | 5,87(1,70) | 5,09(2,92) |
| CutPineapple | fru_0495 | 6,84(2,02) | 3,49(2,77) | 6,43(1,94) | 4,84(2,94) |
| FruitSkewers | fru_0511 | 7,41(1,62) | 4,04(2,75) | 6,29(1,99) | 5,72(2,78) |
| FruitSlicesCut | fru_0553 | 6,37(2,17) | 3,30(2,49) | 5,87(2,02) | 4,51(3,01) |
| FruitSkewers | fru_0593 | 6,68(2,14) | 3,24(2,59) | 6,01(2,33) | 4,91(2,88) |
| ExtendedFruit | fru_0613 | 6,89(1,79) | 3,89(2,64) | 6,09(1,89) | 5,11(2,88) |
| FruitSalad | fru_0670 | 7,65(1,47) | 3,87(2,83) | 6,57(1,95) | 5,71(2,53) |

| Description | Pic  Code | Valence  Mean (SD) | Arousal  Mean (SD) | Dominance  Mean (SD) | Craving  Mean (SD) |
| --- | --- | --- | --- | --- | --- |
|  |  |  |  |  |  |
| FruitSalad | fru_0683 | 7,12(1,90) | 3,99(2,67) | 6,25(2,03) | 5,72(2,71) |
| ExtendedFruit | fru_1902 | 6,80(2,22) | 3,69(2,47) | 5,95(2,37) | 4,87(2,86) |
| CutPineapple | fru_5515 | 7,12(1,64) | 3,77(2,61) | 6,46(2,13) | 5,35(2,66) |
| FruitSlicesCut | fru_5595 | 6,56(2,15) | 3,57(2,73) | 6,17(2,20) | 5,18(2,91) |
| CutPineapple | fru_5772 | 6,35(2,14) | 2,86(2,41) | 5,66(2,10) | 3,97(2,88) |
| Strawberry | fru_5782 | 7,17(2,04) | 4,03(2,81) | 6,29(2,25) | 5,12(3,00) |
| Strawberry | fru_5795 | 7,27(1,93) | 3,49(2,67) | 6,19(2,01) | 5,25(3,01) |
| ExtendedFruit | fru_5833 | 7,31(1,89) | 3,51(2,59) | 6,17(2,20) | 5,06(2,97) |
| Crepes | sug_0013 | 7,86(1,61) | 5,08(2,77) | 6,34(2,15) | 6,64(2,50) |
| Waffles | sug_0014 | 7,43(1,89) | 4,52(2,78) | 6,40(2,19) | 6,03(3,01) |
| Waffles | sug_0018 | 7,79(1,48) | 5,00(2,99) | 6,47(2,34) | 6,63(2,64) |
| Donuts | sug_0043 | 7,31(2,25) | 3,80(2,87) | 5,85(2,37) | 6,10(2,88) |
| Waffles | sug_0072 | 7,58(2,40) | 4,32(3,01) | 6,31(2,16) | 6,51(2,91) |
| Candies | sug_0083 | 7,93(1,95) | 5,03(3,10) | 6,72(2,15) | 6,54(2,92) |
| Candies | sug_0096 | 7,81(1,54) | 4,64(2,74) | 6,34(2,12) | 5,88(2,87) |
| Candies | sug_0099 | 7,65(1,71) | 4,37(2,84) | 6,21(2,18) | 5,81(2,83) |
| Candies | sug_0101 | 7,62(2,17) | 4,11(2,92) | 6,20(2,46) | 5,90(2,91) |
| IceCream | sug_0112 | 7,24(1,93) | 3,79(2,88) | 6,03(2,25) | 5,46(2,92) |
| IceCream | sug_0113 | 7,69(1,44) | 4,30(2,90) | 6,34(1,93) | 6,12(2,72) |
| IceCream | sug_0116 | 6,10(1,86) | 3,25(2,68) | 6,04(2,09) | 3,89(2,80) |

| Description | Pic  Code | Valence  Mean (SD) | Arousal  Mean (SD) | Dominance  Mean (SD) | Craving  Mean (SD) |
| --- | --- | --- | --- | --- | --- |
|  |  |  |  |  |  |
| IceCream | sug_0135 | 7,71(1,59) | 4,40(2,88) | 6,56(1,96) | 6,03(2,77) |
| Crepes | sug_0141 | 7,90(1,36) | 4,62(2,86) | 6,64(2,06) | 6,52(2,58) |
| Pastries | sug_0147 | 6,84(2,00) | 3,91(2,67) | 6,31(1,88) | 5,18(2,72) |
| Donuts | sug_0150 | 7,40(1,82) | 4,00(2,70) | 6,38(2,06) | 6,15(2,78) |
| Pastries | sug_0151 | 6,90(2,02) | 3,64(2,64) | 5,99(2,08) | 4,91(3,09) |
| Donuts | sug_0152 | 7,88(1,40) | 4,91(3,00) | 6,86(1,68) | 6,35(2,77) |
| Donuts | sug_0157 | 7,74(1,51) | 4,74(3,01) | 6,58(1,98) | 6,55(2,46) |
| Pastries | sug_0166 | 6,81(1,96) | 3,80(2,84) | 6,17(2,10) | 5,33(3,01) |
| Pastries | sug_152. | 7,09(1,70) | 3,77(2,60) | 6,17(1,97) | 4,80(2,84) |
| Crepes | sug_4009 | 7,27(1,78) | 4,30(3,00) | 6,51(2,06) | 6,00(3,02) |
| Crepes | sug_4011 | 7,08(2,29) | 4,00(2,81) | 5,92(2,31) | 6,00(3,04) |
| Waffles | sug_4421 | 8,25(1,34) | 5,16(2,95) | 6,79(2,03) | 7,22(2,39) |
| ColdSoup | veg_0002 | 5,83(2,28) | 3,00(2,47) | 6,24(2,21) | 3,89(3,00) |
| VegetableSkewer | veg_0005 | 5,13(1,90) | 2,41(1,74) | 5,29(2,04) | 2,93(2,49) |
| TomatoSalad | veg_0011 | 5,73(2,18) | 2,70(2,24) | 5,25(2,02) | 3,70(2,78) |
| AvocadoSalad | veg_0012 | 5,68(1,76) | 2,66(1,99) | 5,30(1,98) | 3,42(2,65) |
| ColdSoup | veg_0048 | 5,35(1,99) | 2,76(2,30) | 5,20(2,35) | 3,61(2,90) |
| AvocadoSalad | veg_0070 | 5,88(2,04) | 2,71(2,30) | 5,90(1,94) | 3,39(2,85) |
| GrilledVegetables | veg_0079 | 5,48(2,01) | 2,97(2,46) | 5,25(2,03) | 3,50(2,87) |
| VegetableSkewer | veg_0085 | 5,91(1,85) | 2,75(2,20) | 5,90(1,95) | 3,22(2,62) |

| Description | Pic  Code | Valence  Mean (SD) | Arousal  Mean (SD) | Dominance  Mean (SD) | Craving  Mean (SD) |
| --- | --- | --- | --- | --- | --- |
|  |  |  |  |  |  |
| VegetableSkewer | veg_0092 | 5,12(1,84) | 2,54(2,29) | 5,27(2,12) | 2,85(2,46) |
| TomatoSalad | veg_0109 | 6,20(1,93) | 3,13(2,37) | 6,20(2,34) | 4,44(3,03) |
| ColdSoup | veg_0113 | 5,51(1,91) | 2,36(1,85) | 5,46(1,84) | 3,64(2,68) |
| TomatoSalad | veg_0114 | 6,35(2,12) | 3,01(2,54) | 5,91(1,76) | 3,87(2,84) |
| VegetableSkewer | veg_0118 | 5,11(2,31) | 2,66(2,14) | 5,59(2,30) | 3,42(2,66) |
| MixedSalad | veg_0125 | 6,12(2,06) | 3,29(2,65) | 6,19(2,31) | 4,15(3,09) |
| AvocadoSalad | veg_0133 | 5,51(1,98) | 2,49(2,24) | 5,46(1,95) | 3,08(2,59) |
| MixedSalad | veg_0135 | 6,07(1,97) | 2,57(2,25) | 6,21(1,87) | 3,63(2,70) |
| AvocadoSalad | veg_0148 | 5,37(1,87) | 2,65(2,06) | 5,77(2,35) | 3,51(2,71) |
| MixedSalad | veg_0152 | 5,67(1,92) | 2,95(2,21) | 5,36(2,27) | 3,59(2,72) |
| GrilledVegetables | veg_0186 | 5,24(2,17) | 2,69(2,15) | 5,49(2,12) | 3,41(2,71) |
| GrilledVegetables | veg_0198 | 5,60(2,01) | 3,23(2,22) | 5,61(2,13) | 4,31(2,76) |
| MixedSalad | veg_0281 | 5,82(1,92) | 2,94(2,11) | 5,81(2,09) | 3,59(2,67) |
| ColdSoup | veg_0455 | 5,97(2,22) | 2,88(2,33) | 5,85(2,07) | 3,87(3,01) |
| TomatoSalad | veg_0725 | 5,61(2,01) | 2,96(2,32) | 5,34(2,14) | 3,79(2,73) |
| GrilledVegetables | vegofood | 5,77(2,06) | 2,74(2,40) | 5,96(1,99) | 3,33(2,88) |
